# Diverticular Abscess Complicating Pregnancy at 18 Weeks’ Gestation in a 30-Year-Old Female: A Case Report

**DOI:** 10.7759/cureus.50590

**Published:** 2023-12-15

**Authors:** Nathan K Louie, Bradley Champagne

**Affiliations:** 1 Colorectal Surgery, William Carey University College of Osteopathic Medicine, Hattiesburg, USA; 2 Colorectal Surgery, Our Lady of Lourdes Regional Medical Center, Lafayette, USA

**Keywords:** ptb: preterm birth, case report, lower anterior resection with ileostomy, ct guided drainage, pregnancy, diverticulitis

## Abstract

The co-occurrence of diverticulitis with pregnancy is incredibly rare and the management of recurrent complicated diverticulitis may not be feasible in a pregnant patient. Adding cases to the incredibly sparse literature may highlight similarities and create potential recommendations for at-risk populations. We present a case of a female at 18 weeks’ gestation who presented with left lower quadrant pain. The initial physical exam and clinical findings revealed severe abdominal tenderness without signs of generalized peritonitis, leukocytosis with predominant neutrophils, and fundal height with confirmatory ultrasonography of intrauterine pregnancy. The main diagnosis was diverticulitis complicated by an abscess and pregnancy, confirmed with CT imaging. The initial intervention was IV antibiotics and bowel rest; however, with each subsequent discharge, she returned to the emergency department with worsening symptoms. Eventually, at 28 weeks, she was escalated to IV meropenem, CT-guided drainage of the abscess, and preterm vaginal delivery at 28 weeks, with a lower anterior resection and diverting ileostomy at six weeks postpartum. She is currently being followed outpatient with improvement in pain, meaningful healthy weight gain, and a healthy newborn child. While management of diverticulitis is generally straightforward, severe presentations like this, even when compared to existing literature, suggest traditional notions of contraindications and risks may not fully apply. Timing and management of recurrent diverticulitis in pregnancy necessitate further research to establish comprehensive guidelines tailored to these at-risk populations.

## Introduction

Diverticulitis, an inflammatory condition affecting colonic diverticula, is a relatively common gastrointestinal ailment in the general population [1,2]. Its severity is broad but commonly presents with signs of inflammation and left lower quadrant pain due to its propensity to affect the sigmoid colon [3-5].

Although the incidence of diverticular disease has seen an increase in younger populations [1,2,6,7], the co-occurrence of diverticulitis and pregnancy is incredibly rare, with only around a dozen documented case reports and two observational studies [8-11], and may often be overlooked when evaluating an acute abdomen in the obstetric population [12]. Furthermore, one study reviewing a 20-year period of pregnancies within an obstetric department reported an incidence of one in 6000 pregnancies [13], but these cases included small bowel diverticular diseases like Meckel’s diverticulitis. The convergence of these two distinct clinical scenarios can be particularly complex and requires careful consideration to optimize both maternal and fetal outcomes. As such, diverticulitis in pregnancy presents a unique and intricate medical challenge, demanding nuanced decision-making and a multidisciplinary approach to care regarding fetal and maternal outcomes. This was possible because the patient was managed in a large regional medical center. Additionally, the SCARE and CARE Checklist has been completed by the authors for this case report and attached as online supplementary material (Appendices).

## Case presentation

The patient is a 30-year-old Caucasian female, 18 weeks’ gestation, with a history of diverticulosis. The patient initially reported a history of left lower quadrant pain and nausea. The pain was described as sharp/stabbing without radiation, severe, and sudden for 16 hours. She was referred from an outside hospital and had already been started on IV antibiotics and pain control. Ultrasound and CT without contrast from the referring hospital revealed an intrauterine pregnancy at 18 weeks’ gestation and a left sigmoid diverticular abscess.

On admission, the patient was alert but in acute distress with an ill appearance. Her vitals were temperature 100.1 °F (37.8 °C), heart rate 118, respiratory rate 26, blood pressure 140/77, and O2 99%. Mucous membranes dry, left lower quadrant (LLQ) abdominal tenderness, guarding, and hypoactive bowel sounds but without generalized peritoneal findings. Table 1 reveals the initial abnormal tests and Table 2 is a timeline of the relevant hospital encounters.

**Table 1:** Abnormal Results of Initial Diagnostic Laboratory Tests CRP - C-reactive protein

Test	Unit	Value	Normal range
White Blood Cells	1000/µl	14.9	3.5 - 10.8
Absolute Neutrophils	1000/µl	13.6	1.8 - 7.7
Percent Neutrophils	%	91	50 - 70
Absolute Lymphocytes	1000/µl	0.8	1.0 - 4.8
Percent Lymphocytes	%	5	18 - 42
Blood urea nitrogen	mg/dL	4	7 - 20
Co2	mmol/L	20	23 - 29
Total calcium	mg/dL	8.4	8.8 - 10.6
Protein	g/dL	5.8	6 - 8.3
Albumin	g/dL	2.8	3.5 - 5.0
Quantitative CRP	mg/L	90.2	< 3.0
Procalcitonin	ng/mL	17.61	< 0.1
Hemoglobin	g/dL	9.2	12.0 - 16.0
Hematocrit	%	27.4	36.0 - 46.0
Mean corpuscular volume	fL	61	80 - 100
Red cell distribution width	%	17.1	35.1 - 46.3

**Table 2:** Timeline of Relevant Data from Episodes of Care CT - Computer Tomography, IV - Intravenous, LLQ - Left Lower Quadrant, NGT- Nasogastric Tube, MRI - Magnetic Resonance Imaging, PO - Per Os, NICU - Neonatal Intensive Care Unit, LAR - Lower Anterior Resection

Date	Key Events
Day 1	1st Emergency department visit. Initial symptoms were left lower quadrant pain and nausea. Ultrasound revealed an 18-week fetus. CT without contrast suggested a left sigmoid diverticular abscess. The patient was started on IV antibiotics, pain control, and antiemetics.
Day 2	Interventional radiology did not consider CT drainage due to the position of the abscess relative to the uterus and placenta. IV antibiotics treatment showed improvement. The patient was discharged on PO antibiotics.
Day 3	2nd emergency department visit. Presenting symptoms were left lower quadrant pain but with new blood in stool. Repeat CT showed a 3 cm abscess. The patient started on IV antibiotics. This CT image and all subsequent CT images performed utilize dose modulation and/or weight-based dose reduction when appropriate to reduce radiation dose to the patient as low as reasonably achievable.
Day 5	Flexible sigmoidoscopy was performed. Revealed small diverticuli with purulent fluid and mild erythema, rectosigmoid polyps, but otherwise normal. Polyps were removed.
Day 7	Discharged on PO antibiotics with stable labs: minimally elevated WBCs. However, mild LLQ tenderness persisted.
Day 16	3rd emergency department visit. The presenting symptom was lower abdominal pain and severe abdominal distention. Labs showed significant leukocytosis (20.7 1000/mL). MRI imaging suggested enteritis of the Jejunum. The patient was started on IV antibiotics.
Day 17	The patient's infection continued to worsen with leukocytosis peaking at 24.5 1000/mL (neutrophils abs 21.3 1000/mL, neutrophils 87%), Na+ 132 mEq/L, CO2 16 mmol/L, Glucose 109 mg/dL. The patient was started on meropenem and an NGT was placed.
Day 18	CT imaging suggested concern for rectal perforation with small bowel ileus compared to MRI. The patient's status mildly improved. CT-guided drainage was recommended but emergency surgery was discussed for potential complications.
Day 19	Leukocytosis began to resolve. Proceeded with CT-guided drain placed with no complications. Leukocytosis continued to resolve, and the patient began to ambulate. Meropenem was continued for 2 weeks.
Day 25	Discharged with PO antibiotics. The patient's diet was advanced, the pain was controlled, and the drain was in place.
Day 31	1st office visit. The patient had no tenderness along the incision site, <5cc drainage, mild purulent, only flush returns. The drain was removed. Left lower quadrant pain was not resolved but was manageable.
Day 45	2nd office visit. MRI was performed two days prior with no significant changes. No tenderness, no drainage from previous drain site. Left lower quadrant pain is still present but manageable.
Day 64	The patient delivered a 28-week neonate vaginally. The neonate was placed in the NICU.
Day 88	4th emergency department visit. The patient has 6 day history of constipation and lower abdominal pain. The patient was given Miralax, had multiple bowel movements, and was discharged on PO antibiotics for diverticular flares.
Day 112	3rd office visit. The patient continues to have abdominal pain and poor appetite. Options for meaningful resolution of diverticulitis discussed at length: Laparoscopic vs open LAR discussed. Her child was discharged from NICU and at patient's home.
Day 134	Lower anterior resection was performed. Severe scarring and adhesions. Converted to an open procedure, ureteral stent incision and drainage of the pelvis, drain placement, and diverting ileostomy.
Day 154	Post-operative visit. The patient was no longer in pain, and the stoma was functioning.
Day 169	Post-operative visit 2. The patient was doing well, and regaining weight. The patient's incision has healed, the stoma was healthy, and the patient no longer possesses diverticular pain.

Diagnostic assessment and therapeutic interventions

**Figure 1:**
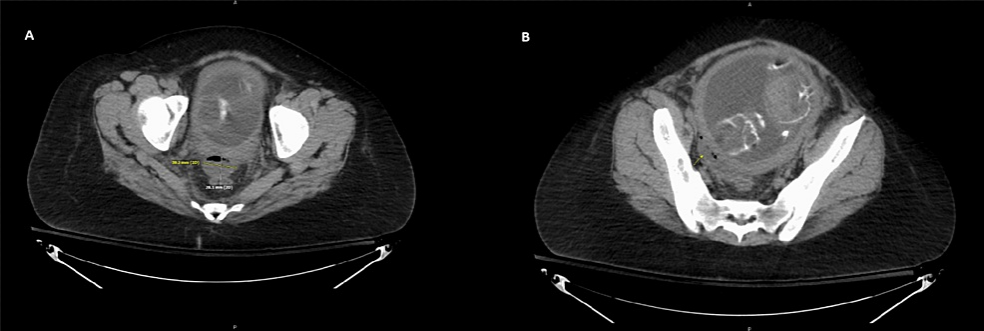
Confirmatory CT Imaging without Contrast of Sigmoid Diverticular Abscess CT scans used dose modulation, iterative reconstruction, and/or weight-based dosing to reduce radiation dose to as low as reasonably achievable. Impression: A: 2.8 x 3.9 cm fluid and gas collection within the rectal pouch of Douglas tracking along the right posterior lateral and superior uterus. B: Sigmoid colonic inflammatory changes and ascites when compared to previous imaging. Prominent gas and stool-filled diverticulum noted extending along the sigmoid antimesenteric border. No gross pneumoperitoneum.

She was ultimately diagnosed with diverticulitis complicated by a 3 cm sigmoid abscess, Figure 1, and a singleton 18-week fetus with no signs of subchorionic hemorrhage or other abnormalities. CT-guided drainage was not considered during this ED visit due to potential complications secondary to the proximity of the abscess to the placenta. She was placed on IV piperacillin-tazobactam, hydromorphone, and fluids and was discharged once her pain and infection were at manageable levels.

**Figure 2:**
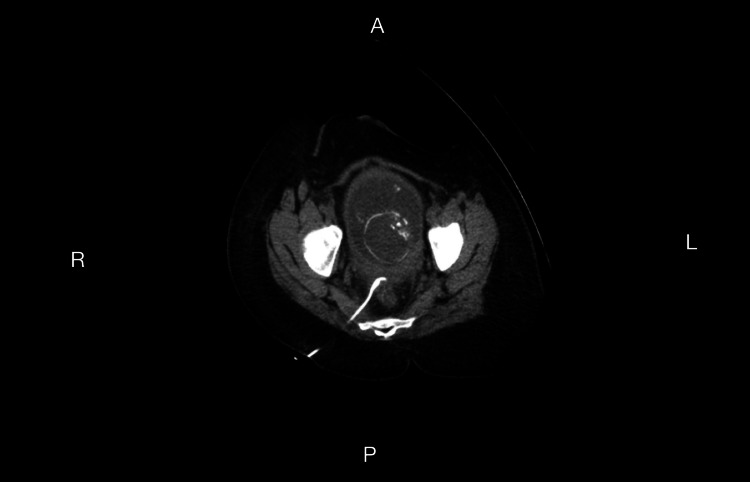
CT-Guided Drain Placement Impression: CT-guided right 8 French transgluteal drain placement into rectouterine pouch fluid collection.

**Figure 3:**
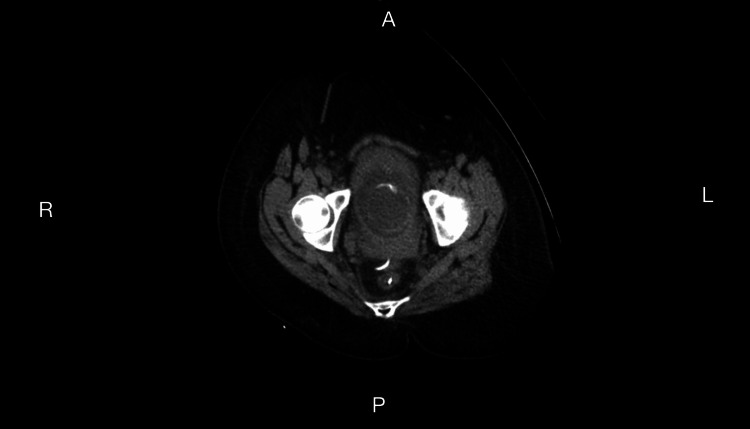
Drain Visualized in Abscess Impression: CT-guided right 8 French transgluteal drain placement into rectouterine pouch fluid collection.

However, over the subsequent weeks, she had multiple flares, as outlined in Table 2. Ultimately, the discussion for CT-guided drainage of the now-confirmed sigmoid abscess was discussed with the patient and her family as a preintervention step in managing her diverticulitis. Furthermore, given the lack of meaningful resolution of her diverticulitis, the option of a partial colectomy was discussed; however, this was once she was well into her postpartum period or in the event of an emergency following her CT-guided drainage at 20 weeks (Figures 2, 3). 

Once her diverticulitis flares were controlled with IV antibiotics, which eventually escalated to meropenem, and the baby was delivered and out of the Neonatal Intensive Care Unit (NICU), she underwent lower anterior resection of her colon at six weeks postpartum.

**Figure 4:**
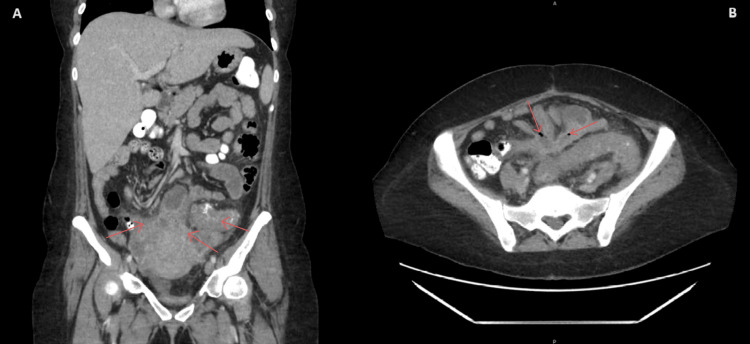
Abdominal and Pelvic CT with IV Contrast Imaging before Lower Anterior Resection Impression: A: Severe diffuse sigmoid colon wall thickening which could reflect colitis or diverticulitis, hyperdensity extrinsic to the sigmoid colon. Pelvic small bowel inflammatory changes with wall thickening, tethering, and areas of distension. B: Small pelvic air-fluid collection suspicious for an interloop abscess on axial image on the right. Diverticulitis may be classified as Hinchey/Kaiser Ib according to CT imaging impression.

A laparoscopic approach was performed, via a median infraumbilical incision. However, after a safe entrance to the abdomen was obtained, pelvic inspection revealed multiple loops of bowel scarred into the pelvis, initially suggested by CT imaging in Figure 4, thus making the operation unsafe for a laparoscopic approach. The median incision was enlarged, and the procedure was converted into an open approach. The multiple loops of small bowel were dissected from their attachments into the site of the pelvis abscess to mobilize them to expose the colon. Once the colon was mobilized, dissection into the pelvis revealed a significant hydroureter which prompted an intraoperative urological consult and stent placement. Following stent placement, further dissection revealed the extent of scar tissue encapsulating a majority of the rectum, vaginal, uterus, the entirety of the sigmoid colon, and a distal portion of the descending colon. Once the scar tissue was adequately dissected and the inflamed colon was separated from the healthy colon, an end-to-end anastomosis from the healthy proximal colon to the rectum was achieved. However, due to the extent of the inflammation and infection, this was considered high risk, and a diverting ileostomy was put in place along with a 19 French black drain.

Pathology revealed 22.5 cm in length and 2.2 cm in diameter sigmoid and rectum with multiple diverticula and peri-diverticular inflammation, benign pseudocyst in subserosa with peri-cystic inflammation, negative for dysplasia/malignancy, and a single benign reactive lymph node that was negative for malignancy.

The patient was admitted and placed on IV piperacillin-tazobactam 4.5 g 100 mL run in at 25 mL/hr with pain management rotating on an as-needed basis between ketorolac 30 mg IV and hydromorphone 0.5 mg IV, and oxycodone-acetaminophen 325 mg by mouth (PO) once oral feeding was tolerated.

Postoperative monitoring confirmed the stability of the patient’s vital signs. Her general condition had improved, and the patient was discharged four days after surgery, afebrile. She had a stoma output of 200 cc, drain output of 100 cc of serosanguinous fluid, and urine output of 650 cc. Incisions were well healed. She was discharged on amoxicillin-clavulanate 125 mg twice daily PO for 10 days and oxycodone-acetaminophen 325 mg every six hours/as needed PO for seven days. She followed up three weeks later no longer in pain, her stoma functioning well, and regaining weight. Her child is healthy and doing well.

## Discussion

Early in training, diverticulitis is taught as a quintessential gastrointestinal diagnosis. However, when paired with other comorbid conditions and, in this case, several potential contraindications in management, it is important to realize the extent to which risk-benefit analysis plays a more instructive role than the general algorithms for treatment of a common and increasingly prevalent gastrointestinal disease.

This case of diverticulitis in pregnancy is only one of a few cases, each with significant differences concerning both the mother’s health and the fetal status. It is these differences that provide both the strengths and limitations of this case report. It is unlikely a case with these exact circumstances will occur again. Therefore, a major limitation of this case report is that these diagnostic steps and treatment options were tailored for this specific case. However, the strength is that should a similar case present in the future, this along with several other cases will illuminate more patterns in better ways to care for diverticulitis in pregnancy. Furthermore, this case resulted in positive outcomes both for the newborn and the mother, which may inform future decisions regarding diagnostic and treatment considerations compared to other case reports.

As mentioned earlier, the existing literature on diverticulitis during pregnancy is notably limited. Moreover, even within comparable instances of sigmoid diverticulitis, the complications and gestational age varied. A study by Kechagias et al. conducted a systematic review revealing all documented cases of colonic diverticulitis in pregnancy amounting to just 12 cases [8]. In one case, the patient was at 33 weeks’ gestation and experienced only one episode of diverticular inflammation before undergoing delivery and subsequent resection due to small bowel obstruction [10]. Furthermore, The American College of Obstetricians and Gynecologists (ACOG) published the ACOG committee opinion number 723, which is supported by the American College of Radiology and the American Institute of Ultrasound in Medicine [14]. The outcomes of this case report and similar literature provide evidence to support this committee's opinion.

## Conclusions

Although diagnosis and treatment of diverticular disease have been well documented and should serve as an initial guide when making initial considerations, management of complications of diverticulitis, such as abscesses and recurrent flares, should be managed with drainage when possible and frequent follow-up until delivery. Resection should be considered in patients where inflammation, pain, or other complications do not resolve in the postpartum period.

The complex decision-making regarding induction of preterm fetuses for maternal beneficence. CT-guided drainage of the abscess was necessary to prevent further perforation and worsening of infection, possibly leading to peritoneal sepsis. In this case, risk-benefit truly takes on a new meaning and is more akin to which risk is the patient willing to take: fetal demise or a possible worsening of the diverticulitis until the sigmoid abscess completely perforates.

Timing and management of recurrent diverticulitis in pregnancy necessitate further research to establish comprehensive guidelines tailored to these at-risk populations.

## Figures and Tables

**Figure 5:**
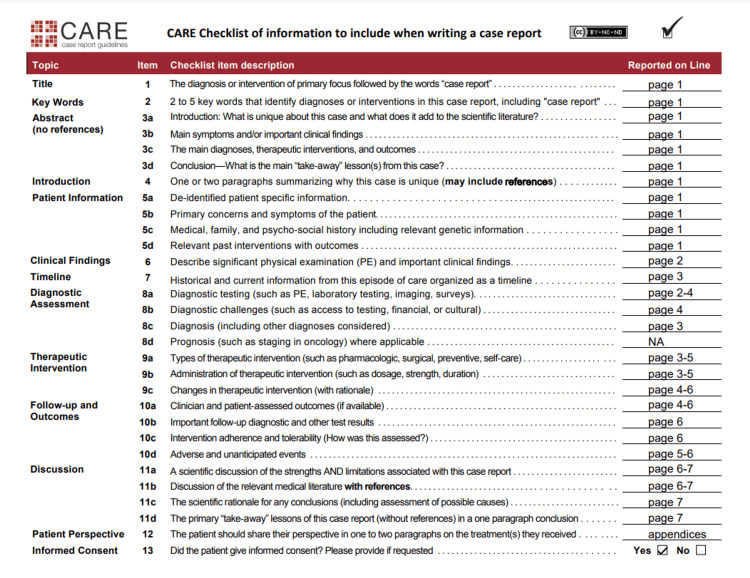
CARE Guideline

**Figure 6:**
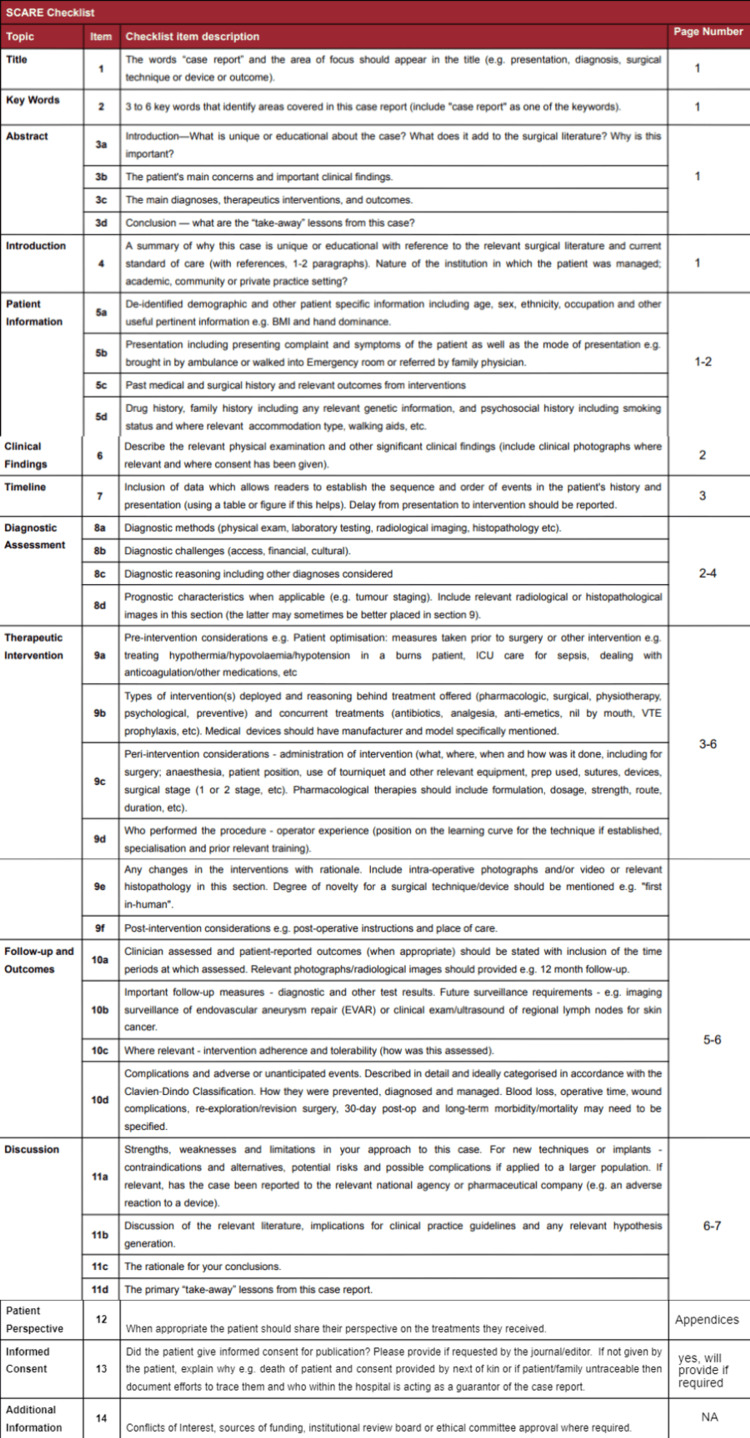
SCARE Guideline

**Figure 7:**
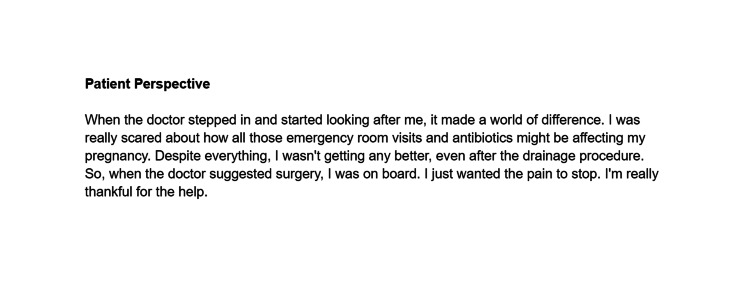
Patient Perspective The patient's perspective was paraphrased from follow-up encounters post-operatively through discussions with the attending colorectal surgeon.
